# *TCF7L2* polymorphisms and the risk of schizophrenia in the Chinese Han population

**DOI:** 10.18632/oncotarget.15603

**Published:** 2017-02-22

**Authors:** Lijun Liu, Jingjie Li, Mengdan Yan, Jing Li, Junyu Chen, Yi Zhang, Xikai Zhu, Li Wang, Longli Kang, Dongya Yuan, Tianbo Jin

**Affiliations:** ^1^ Key Laboratory of Molecular Mechanism and Intervention Research for Plateau Diseases of Tibet Autonomous Region, School of Medicine, Xizang Minzu University, Xianyang, Shaanxi 712082, China; ^2^ Key Laboratory of High Altitude Environment and Genes Related to Diseases of Tibet Autonomous Region, School of Medicine, Xizang Minzu University, Xianyang, Shaanxi 712082, China; ^3^ Key Laboratory for Basic Life Science Research of Tibet Autonomous Region, School of Medicine, Xizang Minzu University, Xianyang, Shaanxi 712082, China; ^4^ Key Laboratory of Resource Biology and Biotechnology in Western China (Northwest University), Ministry of Education, School of Life Sciences, Northwest University, Xi’an, Shaanxi 710069, China; ^5^ Inner Mongolia Medical University, Hohhot, Inner Mongolia, 010050, China

**Keywords:** *TCF7L2*, schizophrenia, SNP, case-control

## Abstract

Single nucleotide polymorphisms (SNPs) in *TCF7L2* (Transcription Factor 7-Like 2) reportedly affect susceptibility to schizophrenia (SCZ). We examined the association between *TCF7L2* polymorphisms and SCZ susceptibility in a Chinese Han population. Six SNPs were genotyped in 499 SCZ patients and 500 healthy individuals, after which their associations with SCZ were evaluated using the Chi-squared test and genetic model analyses. We observed that the allele A of rs12573128 is associated with an increased SCZ risk (odds ratio [OR] = 1.33, 95% confidence interval [CI]: 1.08-1.63, *P* = 0.006, adjusted *P* = 0.030). The AA genotype of rs12573128 was associated with a higher SCZ risk than the GG genotype, before and after adjustment for sex and age (adjusted OR = 2.97, 95% CI: 1.49-5.92, *P* = 0.002). In addition, SNP rs12573128 was associated with 1.47-fold, 2.64-fold and 1.50-fold increases in SCZ risk of in dominant, recessive and additive model, respectively (adjusted OR = 1.47, 95% CI = 1.09-1.99, *P* = 0.012; Bonferroni adjusted *P* = 0.030). adjusted OR = 2.64, 95% CI = 1.34-5.18, *P* = 0.005 and adjusted OR = 1.50, 95% CI = 1.17-1.93, *P* = 0.002, respectively). These results suggest rs12573128 is significantly associated with an increased risk of SCZ in the Chinese Han population.

## INTRODUCTION

Schizophrenia (SCZ) is a severe and complex psychiatric disorder with a lifetime risk of approximately 1% among the population worldwide [1]. It is characterized by delusions, hallucinations, disorganization, dysfunction in normal affective responses, and altered cognitive functioning. Although the pathogenesis of SCZ remains unclear, the risk of SCZ is reportedly associated with complex environmental and genetic factors as well as their interaction with each other [2, 3]. Epidemiological studies indicate that the environmental risk factors for SCZ include urbanized area, minority group, cannabis use, developmental trauma, and pregnancy [4]. From family, twin and adoption studies, there is substantial evidence for the importance of the genetic components that influence the susceptibility to schizophrenia [1, 5]. The heritability of SCZ is estimated to be 64%-80% [6].

Recent association studies have shown that single nucleotide polymorphisms (SNPs) in *TCF7L2* (Transcription Factor 7-Like 2) affect the susceptibility to SCZ in both Arab and European populations [7, 8]. It also appears that chromosome 10q is remarkably rich in linkages to SCZ [9]. *TCF7L2* also known as *TCF4* locus on chromosome 10q25.2-25.3 and encodes a high mobility group box-containing transcription factor that plays a key role in the WNT signaling pathway [10]. This should not be confused with transcription factor 4 (*TCF4*), which share the *TCF4* symbol/alias in common with the *TCF7L2* locus. Previous reported that *TCF4* polymorphisms are associated with the risk of SCZ in the Han Chinese [11]. The WNT signaling pathway has been also associated with SCZ [12, 13]. *TCF7L2* is also thought to be a master regulator of glucose homeostasis through effects on proinsulin production and processing [14, 15]. Consistent with that idea, genetic variants of *TCF7L2* are associated with the risk of type 2 diabetes and gestational diabetes mellitus [16–20].

To further explore the relationship between *TCF7L2* polymorphisms and susceptibility to SCZ in a Chinese Han population, we designed a case-control study including 499 SCZ patients and 500 healthy controls to explore the potential associations between *TCF7L2* polymorphisms and SCZ risk in the Chinese Han population. Our results suggest the SNP rs12573128 in *TCF7L2* is associated with an increased risk of SCZ in the Chinese Han population.

## RESULTS

The basic demographic characteristics of study participants are summarized in Table 1. A total of 499 SCZ patients (263 males and 236 females) with a mean age of 36.7 (±13.3) and 500 healthy controls (192 males and 308 females) with a mean age of 50.4 (±7.8) were enrolled. Although the gender and age distributions differed between cases and controls (*P* < 0.001), these two variables were adjusted in the subsequent multivariate unconditional logistic regression analysis to eliminate the residual confounding effects.

**Table 1:** Characteristics of cases and controls

Variable	Case(n=499)	Control(n=500)	*P*
Sex			< 0.001
Male	263 (52.7)	192 (38.4)	
Female	236 (47.3)	308 (61.6)	
Age			< 0.001
yr (mean ± SD)	36.7±13.3	50.4±7.8	

*P* < 0.05 indicates statistical significance.

Six SNPs (rs12573128, rs7081062, rs7081062, rs7081062, rs6585205 and rs290489) in *TCF7L2* were successfully genotyped in both the cases and healthy controls. The sequences of the primers for each SNP polymerase chain reaction (PCR) and single base extension reaction are listed in Table 2. The allele distributions and minor allele frequency (MAF) of the SNPs and results of Hardy-Weinberg equilibrium (HWE) test are shown in Table 3. All six SNPs were in HWE in the control subjects (*P* > 0.05). Comparison of differences in the frequency distributions of alleles between cases and controls using the Chi-squared test and found that allele “A” of rs12573128 was significantly associated with a 1.33-fold increase in risk of SCZ at a 5% level (OR = 1.33, 95% CI: 1.08-1.63, *P* = 0.006, Bonferroni adjusted *P* = 0.030). Comparisons of the SNP genotypes and risk of SCZ are listed in Table 4. The AA genotype of rs12573128 was associated with significantly higher risk of SCZ than the GG genotype both before and after adjustment for age and gender (OR = 2.07, 95% CI: 1.49-5.92, *P* = 0.013; adjusted OR = 2.97, 95% CI: 1.49-5.92, *P* = 0.002, respectively).

**Table 2:** Sequence of oligonucleotide primers used for the analysis of *TCF7L2* polymorphisms

SNP-ID	1st-PCRP	2nd-PCRP	UEP
rs12573128	ACGTTGGATGCAGGTAACT TGCTCAAGAGG	ACGTTGGATGCATTCTTCTG AGGTATGGAC	TGGACTTAAATTAGCT AATTAGG
rs7081062	ACGTTGGATGCCTTTAAGT TCTCTTTCAAG	ACGTTGGATGTGAAGAAGCC AAGAGTTTCC	AGAGTTTCCTGTTAATT AAAAAGA
rs4918789	ACGTTGGATGCAAAAGGCA AGGCGATTTTC	ACGTTGGATGCATGGTGTAC AACTCACACT	TTTGCTCTCTACA CCCTCA
rs3750804	ACGTTGGATGAGAAAGGTG CCAGCTTCAAC	ACGTTGGATGTTTCTGGGGC GGTCGCAGG	aCGCAGGCTGACTAACA
rs6585205	ACGTTGGATGTATTGACCC AACTTGGTCCC	ACGTTGGATGCTCTTGCCAT TCCTGGTTTC	actgGCCATTCCTGGTTTCA TCTAAGT
rs290489	ACGTTGGATGTGCTGTCCC CAGCTTCTTTC	ACGTTGGATGCTTGACCTGT CTTTCCAGGC	TTCCAGGCCCTTCTC

Abbreviations: SNP: Single nucleotide polymorphism; PCRP: Polymerase chain reaction primer; UEP: Unique base extension primer.

Sequences are written in the 5′→3′ (left to right) orientation.

**Table 3:** Allele frequencies in cases and controls and odds ratio estimates for SCZ

SNP-ID	Band	Position	Role	Alleles A/B	MAF		HWE	OR	95% CI		*P*^a^	*P*^b^
					Case	Control						
rs12573128	10q25.2	114730797	Intron	A/G	0.278	0.225	0.200	1.33	1.08	1.63	0.006	0.030
rs7081062	10q25.2	114740745	Intron	G/A	0.252	0.224	0.798	1.17	0.95	1.43	0.142	0.710
rs4918789	10q25.2	114821807	Intron	G/T	0.050	0.039	0.537	1.30	0.85	2.00	0.225	1.125
rs3750804	10q25.2	114833850	Intron	T/C	0.231	0.241	0.541	0.95	0.77	1.17	0.616	3.080
rs6585205	10q25.2	114859164	Intron	T/G	0.459	0.440	1.000	1.08	0.90	1.29	0.395	1.975
rs290489	10q25.3	114907055	Intron	A/G	0.342	0.348	0.554	0.98	0.81	1.17	0.791	3.955

Abbreviations: SNP: Single nucleotide polymorphism; MAF: Minor allele frequency; HWE: Hardy-Weinberg equilibrium; OR: Odds ratio; 95% CI: 95% Confidence interval.

^a^*P* values were calculated using the Chi-Square test.

^b^*P* values were adjusted by Bonferroni correction.

*P* < 0.05 indicates statistical significance.

**Table 4:** Genotypes of the six SNPs and their associations with risk of SCZ

SNP-ID	Genotype	Case(N)	Control(N)	Without adjustment			With adjustment		
				OR	95% CI	*P*	OR	95% CI	*P*
rs12573128	GG	257	295	1.00	-		1.00	-	
	AG	205	185	1.27	0.98-1.65	0.070	1.34	0.98-1.83	0.067
	AA	36	20	2.07	1.17-3.66	0.013	2.97	1.49-5.92	0.002
rs7081062	AA	275	302	1.00	-		1.00	-	
	GA	195	172	1.25	0.96-1.62	0.101	1.30	0.95-1.7	0.101
	GG	28	26	1.18	0.68-2.07	0.556	1.38	0.70-2.73	0.358
rs4918789	TT	448	462	1.00	-		1.00	-	
	GT	50	37	1.39	0.89-2.17	0.143	1.43	0.86-2.39	0.169
	GG	0	1	0.00	-	0.999	0.00	-	0.999
rs3750804	CC	293	285	1.00	-		1.00	-	
	TC	181	189	0.93	0.72-1.21	0.594	0.85	0.62-1.17	0.322
	TT	25	26	0.94	0.53-1.66	0.819	1.08	0.55-2.12	0.815
rs6585205	GG	142	156	1.00	-		1.00	-	
	TG	255	247	1.13	0.85-1.51	0.390	1.02	0.72-1.44	0.903
	TT	101	96	1.16	0.81-1.66	0.431	1.30	0.85-1.99	0.234
rs290489	GG	222	209	1.00	-		1.00	-	
	AG	211	234	0.85	0.65-1.11	0.226	0.85	0.62-1.16	0.302
	AA	65	57	1.07	0.72-1.61	0.730	0.95	0.58-1.55	0.832

Abbreviations: SNP: Single nucleotide polymorphism; OR: Odds ratio; 95% CI: 95% Confidence interval.

*P* values were calculated using the Wald test.

*P* < 0.05 indicates statistical significance.

The results of genetic model analyses (dominant, recessive and additive) using unconditional logistic regression analysis after adjustment with age and gender are summarized in Table 5. We determined that SNP rs12573128 was associated with 1.47-fold, 2.64-fold and 1.50-fold increased risk of SCZ in the dominant, additive and recessive models, respectively (adjusted OR = 1.47, 95% CI = 1.09-1.99, *P* = 0.012; adjusted OR = 2.64, 95% CI = 1.34-5.18, *P* = 0.005 and adjusted OR = 1.50, 95% CI = 1.17-1.93, *P* = 0.002, respectively).

**Table 5:** Genetic model analyses of the association between the SNPs and SCZ with adjustment for age and gender

SNP-ID	Dominant				Recessive				Additive			
	OR	95% CI		*P*	OR	95% CI		*P*	OR	95% CI		*P*
rs12573128	1.47	1.09	1.99	0.012	2.64	1.34	5.18	0.005	1.50	1.17	1.93	0.002
rs7081062	1.31	0.97	1.77	0.080	1.24	0.64	2.44	0.524	1.24	0.97	1.60	0.090
rs4918789	1.41	0.85	2.34	0.185	-	-	-	0.999	1.38	0.84	2.26	0.209
rs3750804	0.88	0.65	1.19	0.403	1.15	0.60	2.24	0.673	0.93	0.73	1.20	0.593
rs6585205	1.09	0.79	1.51	0.588	1.28	0.88	1.85	0.192	1.13	0.91	1.39	0.271
rs290489	0.87	0.64	1.17	0.347	1.03	0.64	1.65	0.902	0.93	0.74	1.16	0.521

Abbreviations: SNP: Single nucleotide polymorphism; OR: Odds ratio; 95% CI: 95% Confidence interval.

*P* < 0.05 indicates statistical significance.

## DISCUSSION

The present study of 999 participants (499 SCZ patients and 500 healthy controls) was designed to investigate whether *TCF7L2* SNPs previously associated with the risk of type 2 diabetes and SCZ in an Arab population are associated with the risk of SCZ in a Chinese Han population. Our results indicated that the SNP rs12573128 in *TCF7L2* increases risk of SCZ.

*TCF7L2* is a reportedly transcription factor and a key component of the canonical WNT signaling pathway that regulates cell proliferation and differentiation [21, 22]. This pathway plays role in central nervous system (CNS) development [23], and is associated with SCZ [13]. It was previously observed that some *TCF7L2* polymorphisms were associated with an increased risk of SCZ in Arab and European populations [7, 8], though the molecular and cellular mechanisms through which *TCF7L2* act in this regard remain unclear. In the adult mouse, levels of *TCF7L2* expression are high in thalamus and tectum, with lower expression levels in the hypothalamus and other areas. This suggests *TCF7L2* may play a key role in mediating autonomic homeostasis [24]. Moreover, *TCF7L2* expression in the CNS is characterized by the presence of several splice variants, expression of which in mice differs among postmitotic neurons, immature neural precursors and intestinal epithelum [25]. Furthermore, a uniquely spliced *TCF7L2* form of may share neuroendocrine functions important for human brain, pancreatic islets and gut [26]. This is consistent with the finding that SCZ patients are at increased risk of comorbidities such as type 2 diabetes, and that TCF7L2 is associated with type 2 diabetes [7, 8]. We excluded patients with comorbidities from the study so that our results would not be affected by comorbidities.

The present study is generally consistent with the earlier finding that rs12573128 was associated with SCZ risk in an Arab population [8]. Our result suggests SNP rs12573128 affects WNT signaling to impact essential functions of *TCF7L2* during neural development, and may also impact the maturity of oligodendrocyte progenitor cells associated with the pathogenesis of SCZ.

This study has some potential limitations. First, the sample size is relatively small, and the participants included only a Chinese Han population. Second, although several confounders were adjusted for in the statistical analyses, we could not completely eliminate the potential influences of these factors on the results. Third, we conducted no functional analyses of the genetic variants in *TCF7L2*. In future studies with larger samples, functional characterizations will be important for validation of our findings.

In conclusion, our results indicate that SNP rs12573128 in *TCF7L2* was is associated with an increased risk of SCZ in a Chinese Han population, and could potentially serve as a clinically important pre-diagnostic marker.

## MATERIALS AND METHODS

### Ethics statement

The study was approved by the Ethics Committee of the Psychiatric Hospital of Xi’an and Xizang Minzu University (Shaanxi, China), and complied with the Declaration of Helsinki. All participants gave written informed consent prior to participation in the study.

### Study participants

We recruited 499 SCZ patients form the Psychiatric Hospital of Xi’an, Shanxi province between April 2011 and January 2015, and the sample set is same as the former study on association between TCF4 polymorphisms and SCZ risk [11]. The patients were interviewed by at least two experienced senior psychiatrists and diagnosed strictly according to the criteria of the DSM-IV (Diagnostic and Statistical Manual of Mental Disorders, Fourth Edition) based on the Chinese version of the SCID-1 (Structured Clinical Interview for DSM-IV Axis I Disorders). In addition, 500 healthy controls were randomly selected from among unrelated volunteers recruited through advertisements in Xi’an city and its vicinity. These controls were screened using the SCID-I, non-patient edition. The inclusion and exclusion criteria were as follows. (1) All participants were genetically unrelated ethnic Han Chinese whose ancestors had lived in the region for at least three generations. (2) SCZ patients who had comorbidity, such as diabetes mellitus, hypertension or any endocrine disorder were excluded. (3) All the SCZ patients were recruited without age, sex, or disease stage restriction. (4) Healthy subjects were excluded if they had a family history of psychiatric disorder, or a history of hypothyroidism, uncontrolled hypertension or diabetes, or significant drug or alcohol abuse.

### Clinical data and demographic information

The basic characteristics of all controls, including residential region, ethnicity, sex, age, family history and alcohol intake were collected by well-trained interviewers using a standardized questionnaire. Clinical information about the patients was collected from medical records and pathology reports.

### DNA extraction

We collected 5 mL of peripheral venous blood from each participant using vacutainer tubes containing ethylene diamine tetra-acetic acid (EDTA), which were then stored at −80°C. Genomic DNA was extracted from the blood samples using a GoldMag-Mini Whole Blood Genomic DNA Purification Kit according to the manufacturer's protocol (GoldMag. Co. Ltd., Xi’an, China). DNA concentration and purity were evaluated using a spectrophotometer (NanoDrop 2000; Thermo Fisher Scientific, Waltham, MA, USA).

### SNP selection and genotyping

We selected six SNPs (rs12573128, rs7081062, rs7081062, rs7081062, rs6585205 and rs290489) reportedly associated with the risk of type 2 diabetes [16–19] and SCZ [8]. The MAFs of all of the SNPs were >5% in the HapMap Han Chinese population. PCR and extension primers for the SNPs were designed using the Sequenom MassARRAY Assay Design 3.0 software (Sequenom, San Diego, CA, USA). Genotyping of the SNPs was performed with the Sequenom MassARRAY platform according to the standard instructions recommended by the manufacturer (Sequenom, San Diego, CA, USA) [27]. Sequenom Typer 4.0 software was used for data management and analysis.

### Statistical analyses

The SPSS 19.0 (SPSS Inc., Chicago, IL, USA) and Microsoft Excel (Microsoft Corp., Redmond, WA, USA) were used for statistical analyses. The gender distribution was compared using the Chi-squared test, while the age among between the cases and controls was assessed using Welch's t test. Genotypic frequencies in the controls were tested for departure from the HWE using Firsher's exact test. The allele frequencies of the cases and controls were calculated by use of Chi-squared test/Firsher's exact test, and we performed Bonferroni correction to adjust the *P* values. The genotype frequencies of the cases and controls were calculated by the Wald test. The relative risk was estimated based on odds ratios (ORs) and 95% confidence intervals (CIs) [28]. Genetic model analyses (dominant, recessive and additive) were applied using PLINK software to assess the significance of SNPs [29]. ORs and 95% CIs were calculated using unconditional logistic regression analysis with adjustment for age and gender. All *P*-values presented were two sided, and *P* value < 0.05 was considered statistically significant.

## References

[1] Craddock N, O’Donovan MC, Owen MJ (2005). The genetics of schizophrenia and bipolar disorder: dissecting psychosis. J Med Genet.

[2] Tsuang MT, Stone WS, Faraone SV (2001). Genes, environment and schizophrenia. Br J Psychiatry Suppl.

[3] Cannon TD, van Erp TG, Bearden CE, Loewy R, Thompson P, Toga AW, Huttunen MO, Keshavan MS, Seidman LJ, Tsuang MT (2003). Early and late neurodevelopmental influences in the prodrome to schizophrenia: contributions of genes, environment, and their interactions. Schizophr Bull.

[4] van Os J, Kenis G, Rutten BP (2010). The environment and schizophrenia. Nature.

[5] Cardno AG, Gottesman II (2000). Twin studies of schizophrenia: from bow-and-arrow concordances to star wars Mx and functional genomics. Am J Med Genet.

[6] Thaker GK, Carpenter WT (2001). Advances in schizophrenia. Nat Med.

[7] Hansen T, Ingason A, Djurovic S, Melle I, Fenger M, Gustafsson O, Jakobsen KD, Rasmussen HB, Tosato S, Rietschel M, Frank J, Owen M, Bonetto C (2011). At-risk variant in TCF7L2 for type II diabetes increases risk of schizophrenia. Biol Psychiatry.

[8] Alkelai A, Greenbaum L, Lupoli S, Kohn Y, Sarner-Kanyas K, Ben-Asher E, Lancet D, Macciardi F, Lerer B (2012). Association of the type 2 diabetes mellitus susceptibility gene, TCF7L2, with schizophrenia in an Arab-Israeli family sample. PLoS One.

[9] Alkelai A, Kohn Y, Olender T, Sarner-Kanyas K, Rigbi A, Hamdan A, Ben-Asher E, Lancet D, Lerer B (2009). Evidence for an interaction of schizophrenia susceptibility loci on chromosome 6q23.3 and 10q24.33-q26.13 in Arab Israeli families. American journal of medical genetics Part B, Neuropsychiatric genetics.

[10] Struewing I, Boyechko T, Barnett C, Beildeck M, Byers SW, Mao CD (2010). The balance of TCF7L2 variants with differential activities in Wnt-signaling is regulated by lithium in a GSK3beta-independent manner. Biochem Biophys Res Commun.

[11] Li J, Chen Z, Wang F, Ouyang Y, Zhang N, Yang M, Yan M, Zhu X, He X, Yuan D, Jin T (2016). Polymorphisms of the TCF4 gene are associated with the risk of schizophrenia in the Han Chinese. American journal of medical genetics Part B, Neuropsychiatric.

[12] Singh KK (2013). An emerging role for Wnt and GSK3 signaling pathways in schizophrenia. Clin Genet.

[13] Inestrosa NC, Montecinos-Oliva C, Fuenzalida M (2012). Wnt signaling: role in Alzheimer disease and schizophrenia. Journal of neuroimmune.

[14] Zhou Y, Park SY, Su J, Bailey K, Ottosson-Laakso E, Shcherbina L, Oskolkov N, Zhang E, Thevenin T, Fadista J, Bennet H, Vikman P, Wierup N (2014). TCF7L2 is a master regulator of insulin production and processing. Hum Mol Genet.

[15] Takamoto I, Kubota N, Nakaya K, Kumagai K, Hashimoto S, Kubota T, Inoue M, Kajiwara E, Katsuyama H, Obata A, Sakurai Y, Iwamoto M, Kitamura T (2014). TCF7L2 in mouse pancreatic beta cells plays a crucial role in glucose homeostasis by regulating beta cell mass. Diabetologia.

[16] Chang YC, Chang TJ, Jiang YD, Kuo SS, Lee KC, Chiu KC, Chuang LM (2007). Association study of the genetic polymorphisms of the transcription factor 7-like 2 (TCF7L2) gene and type 2 diabetes in the Chinese population. Diabetes.

[17] Ng MC, Tam CH, Lam VK, So WY, Ma RC, Chan JC (2007). Replication and identification of novel variants at TCF7L2 associated with type 2 diabetes in Hong Kong Chinese. J Clin Endocrinol Metab.

[18] Lin Y, Li P, Cai L, Zhang B, Tang X, Zhang X, Li Y, Xian Y, Yang Y, Wang L, Lu F, Liu X, Rao S (2010). Association study of genetic variants in eight genes/loci with type 2 diabetes in a Han Chinese population. BMC Med Genet.

[19] Sanghera DK, Nath SK, Ortega L, Gambarelli M, Kim-Howard X, Singh JR, Ralhan SK, Wander GS, Mehra NK, Mulvihill JJ, Kamboh MI (2008). TCF7L2 polymorphisms are associated with type 2 diabetes in Khatri Sikhs from North India: genetic variation affects lipid levels. Ann Hum Genet.

[20] Ye D, Fei Y, Ling Q, Xu W, Zhang Z, Shu J, Li C, Dong F (2016). Polymorphisms in TCF7L2 gene are associated with gestational diabetes mellitus in Chinese Han population. Sci Rep.

[21] Smith U (2007). TCF7L2 and type 2 diabetes—we WNT to know. Diabetologia.

[22] Huelsken J, Birchmeier W (2001). New aspects of Wnt signaling pathways in higher vertebrates. Curr Opin Genet Dev.

[23] Backman M, Machon O, Mygland L, van den Bout CJ, Zhong W, Taketo MM, Krauss S (2005). Effects of canonical Wnt signaling on dorso-ventral specification of the mouse telencephalon. Dev Biol.

[24] Lee S, Lee CE, Elias CF, Elmquist JK (2009). Expression of the diabetes-associated gene TCF7L2 in adult mouse brain. J Comp Neurol.

[25] Nazwar TA, Glassmann A, Schilling K (2009). Expression and molecular diversity of Tcf7l2 in the developing murine cerebellum and brain. J Neurosci Res.

[26] Prokunina-Olsson L, Hall JL (2010). Evidence for neuroendocrine function of a unique splicing form of TCF7L2 in human brain, islets and gut. Diabetologia.

[27] Gabriel S, Ziaugra L, Tabbaa D (2009). SNP genotyping using the Sequenom MassARRAY iPLEX platform. Curr Protoc Hum Genet.

[28] Bland JM, Altman DG (2000). Statistics notes. The odds ratio. BMJ.

[29] Purcell S, Neale B, Todd-Brown K, Thomas L, Ferreira MA, Bender D, Maller J, Sklar P, de Bakker PI, Daly MJ, Sham PC (2007). PLINK: a tool set for whole-genome association and population-based linkage analyses. Am J Hum Genet.

